# Supplementary material for: Determining Irradiation Dose in Potato Tubers During Storage Using Reaction-Based Pattern Recognition Method
Source: Foods. 2025 Dec 12;14(24):4285. doi: 10.3390/foods14244285 (PMC12732958; doi:10.3390/foods14244285)
Supplement: Supplementary file 1 [file foods-14-04285-s001.zip › foods-3989100-supplementary.pdf]

Supplementary Information for paper

Determining Irradiation Dose in Potato Tubers During Storage using Reaction-based Pattern Recognition Method

by Yana V. Zubritskaya,[2,3] Anna V. Shik[1], Irina A. Stepanova[1], Sergey A. Zolotov[2,3], Polina Yu. Borshchegovskaya[2,3], Ulyana A. Bliznyuk[2,3], Irina A. Ananieva[1], Alexander P. Chernyaev[1,2], Igor A. Rodin[1,4] and Mikhail K. Beklemishev[1*]

[1] Department of Chemistry, Lomonosov Moscow State University, GSP-1, 1-3 Leninskiye Gory, 119991 Moscow, Russia;
[2] Skobeltsyn Institute of Nuclear Physics, Lomonosov Moscow State University, GSP-1, 1-2 Leninskiye Gory, 119991 Moscow, Russia;
[3] Department of Physics, Lomonosov Moscow State University, GSP-1, 1-2 Leninskiye Gory, 119991 Moscow, Russia;
[4] MIREA - Russian Technological University (Lomonosov Institute of Fine Chemical Technologies), 78 Vernadsky Ave., 119571 Moscow, Russia

Table S1. A fragment of a data table for Agatha variety (2nd day of storage) representing the intensities of the photographic images of the 96-well plate containing reaction mixtures. The actual table contained 26 data columns containing more timepoints and wavelengths of the redox reaction. Observations represent parallel runs for two samples (1 and 2) per dose (Control = 0 Gy, 100 Gy, 1000 Gy).

Observation	Aggregation reaction						Redox reaction		
	660 nm fl	366 nm fl	254 nm fl	R vis	G vis	B vis	366 0 min	366 10 min	366 20 min
Agata Control (1)	49.1	42.3	39.0	156.8	174.4	158.1	19.8	21.1	26.4
Agata Control (1)	40.6	43.3	39.3	160.3	175.5	158.0	17.9	18.5	21.7
Agata Control (1)	51.4	44.5	43.0	151.8	171.9	157.0	18.4	19.3	22.6
Agata Control (1)	45.8	42.4	40.2	158.8	174.0	156.5	19.3	19.6	22.2
Agata Control (1)	52.4	39.4	37.6	155.2	171.7	155.7	17.8	19.0	21.0
Agata Control (2)	43.3	41.1	38.5	156.0	171.6	155.4	17.0	19.0	23.5
Agata Control (2)	47.2	42.3	40.6	153.4	172.0	155.7	17.3	18.4	20.8
Agata Control (2)	57.6	39.3	37.8	149.8	170.2	154.6	17.7	19.2	23.3
Agata Control (2)	51.4	40.7	39.9	148.2	169.7	153.8	18.6	20.8	25.0
Agata Control (2)	43.2	44.2	40.1	159.0	174.5	158.7	18.5	19.2	23.0
Agata 100 Gy (1)	129.4	39.9	41.1	144.9	160.9	139.4	23.1	21.8	22.7
Agata 100 Gy (1)	139.7	42.1	43.5	143.5	162.0	140.8	22.1	20.6	21.0
Agata 100 Gy (1)	121.5	42.0	43.9	148.0	160.2	137.7	22.0	21.3	21.4
Agata 100 Gy (1)	137.5	43.3	45.8	143.6	160.7	138.2	25.2	23.8	24.0
Agata 100 Gy (1)	152.8	39.9	41.5	144.0	165.3	146.7	24.9	23.7	24.4
Agata 100 Gy (2)	137.4	41.3	44.1	150.1	164.8	143.4	24.0	21.5	22.7
Agata 100 Gy (2)	128.6	43.2	46.2	148.6	164.3	143.6	23.8	22.1	22.1
Agata 100 Gy (2)	143.4	42.7	44.7	143.7	162.5	142.2	22.3	21.4	21.5
Agata 100 Gy (2)	123.1	43.1	44.4	154.2	166.1	144.1	24.1	22.3	23.5
Agata 100 Gy (2)	136.5	40.6	44.0	142.7	159.9	137.7	20.9	21.3	21.2
Agata 1000 Gy (1)	130.3	43.9	41.8	146.1	164.4	144.3	23.4	21.9	22.3
Agata 1000 Gy (1)	133.4	42.7	41.5	146.8	164.6	144.2	24.3	23.5	23.0
Agata 1000 Gy (1)	122.8	42.4	40.4	151.7	165.3	144.0	23.8	23.2	23.9
Agata 1000 Gy (1)	126.0	44.6	42.0	152.5	165.6	143.6	26.0	23.5	23.4
Agata 1000 Gy (1)	114.0	44.2	40.5	155.2	166.3	143.2	23.7	22.4	22.5
Agata 1000 Gy (2)	130.0	46.2	47.2	147.1	168.7	147.1	24.3	23.6	23.1
Agata 1000 Gy (2)	120.1	45.3	46.5	152.1	168.0	147.1	24.1	24.2	25.4
Agata 1000 Gy (2)	117.1	47.0	51.3	148.5	169.4	149.3	24.8	24.7	26.1
Agata 1000 Gy (2)	119.7	49.9	52.0	146.4	169.3	147.3	24.7	23.2	24.1
Agata 1000 Gy (2)	126.0	47.5	47.8	146.0	167.0	146.5	23.8	23.1	23.5
